# Intraocular Pressure Measurements by Three Different Tonometers in Children with Aphakic Glaucoma and a Thick Cornea

**Published:** 2014-01

**Authors:** Mohammad Reza Razeghinejad, Ramin Salouti, Mohammad Reza Khalili

**Affiliations:** 1 Poostchi Ophthalmology Research Center, Shiraz University of Medical Sciences, Shiraz, Iran

**Keywords:** Aphakia, Cornea, Intraocular pressure, Tonometry

## Abstract

**Background: **To evaluate the agreement in intraocular pressure (IOP) measurements by Ocular Response Analyzer (ORA) and Tono-Pen XL (TXL) with the Goldmann Applanation Tonometer (GAT) and to examine corneal biomechanical properties in aphakic glaucoma patients with a central corneal thickness (CCT) >600 µ.

**Methods: **Thirty-six eyes of aphakic glaucoma patients (group 1) and 40 eyes of normal children (group 2) were studied. The mean ORA and TXL IOP values were compared with the GAT-IOP values. Regression analyses were used to evaluate the associations between IOP and CCT, corneal hysteresis (CH), and corneal resistance factor (CRF). Bland-Altman plots were used to evaluate the agreement between the tonometers.

**Results: **The mean±standard deviations of the age and male/female ratio were 16.58±5.44 and 15.75±5.04 years and 14/22 and 18/22 in group 1 and group 2, respectively. CCT in group 1 was 651.1±42 and in group 2 was 567.3±32.4. In group 1, the mean TXL (22.4, P=0.004), IOPcc (corneal compensated) (27.8, P=0.005), and IOPg (Goldmann correlated) values (28.1, P<0.0001) were greater than GAT-IOP (20.6). In group 2, only IOPg value (16.4) was higher than GAT-IOP (14.8, P=0.04). IOP reading of all the tonometers were positively and negatively associated with CRF and CH in the multiple regression analysis, respectively.

**Conclusion: **The TXL had a greater agreement with the GAT, and the ORA overestimated IOP in aphakic glaucoma patients. The ORA and TXL seemed to be affected by CH and CRF.

## Introduction

The risk of glaucoma has been determined to be as high as 32% among aphakic children in previous studies.^1^^-^^3^With longer follow-up, the prevalence may be as high as 100%.^4^ Clinical optic nerve head evaluation, gonioscopy, computerized perimetry, and other examinations may be difficult to perform in children. Additionally, the only method of glaucoma treatment for which there is extensive evidence is therapy to decrease intraocular pressure (IOP). Thus, IOP has an important role in the evaluation of aphakic children with glaucoma or at risk for glaucoma.^5^ It has been shown that central corneal thickness (CCT) in these patients is higher than that in their normal peers.^6^^-^^9^It is now recognized that biomechanical properties of the cornea are also important, in addition to the geometric thickness. The study of CCT and corneal biomechanical characters and their effects on the measured IOP using common tonometers in this particular group may assist in our understanding and management of this unique group of patients.

The Goldmann Applanation Tonometer (GAT) is regarded as the reference standard for checking IOP. However, it is common knowledge that the accuracy of the device, that is, its ability to provide a measure of the true IOP, is affected by corneal properties. The Ocular Response Analyzer (ORA, Reichert Ophthalmic Instruments, Inc., Buffalo, New York, USA) is a noncontact device that analyzes corneal biomechanical properties simply and rapidly. Variables obtained by the ORA are corneal-compensated IOP (IOPcc), Goldmann-correlated IOP (IOPg), corneal hysteresis (CH), and corneal resistance factor (CRF). IOPg corresponds to IOP measured with GAT, and IOPcc is thought to be less affected by corneal properties than GAT. The Tono-Pen XL (TXL, Reichert Ophthalmic Instruments, Buffalo, USA) is a portable hand-held instrument. It is based on the Mackay-Marg principle and utilizes micro strain gauge technology. A 1.00 mm transducer tip, covered by a disposable single-use cap, contacts the cornea and displays the average of four independent readings.^10^ It is known that corneal thickness affects the measured IOP.^11^ The ORA has been proposed to measure IOP independent of corneal thickness, and the TXL has been suggested to be less affected by corneal thickness because of its small area of contact with the cornea while measuring IOP.

We sought to determine whether the thick cornea of patients with aphakic glaucoma affects the readings of these tonometers compared to GAT. The primary purpose of our study was to determine the agreement between the measurement of IOP by the TXL (suggested to be less affected by the cornea because of the small area of contact with the cornea while measuring IOP) and ORA (proposed to measure IOP independent of the corneal characters) with GAT, as a standard tonometer, in a group of aphakic glaucoma children with a CCT greater than 600 µ. Secondary objectives were to determine corneal biomechanical properties in this group of patients. Finally, we aimed to find out the effects of CH, CRF, and CCT values on the IOP measurements obtained using the aforementioned tonometers. 

## Patients and Methods

This cross-sectional study was conducted after approval from the local Ethics Committee. Informed consent was obtained from the parents of the enrolled children in the study. We used Power SSC program (version 1.00) (Sample Size Calculator and Power Analysis). With regard to the power of 80% and alpha of 0.05, the sample size was determined to be 76 eyes. The study was conducted from September 2010 to September 2011 in a tertiary eye care hospital in Shiraz, Iran. A full ophthalmic examination was carried out on all the participants, including slit lamp biomicroscopy and fundus slit lamp biomicroscopy using the Volk Superfield lens. In the same visit, all the participants underwent the pachymetry test, and the eligible individuals underwent IOP measurements on another day. 

Group 1 patients were selected among a consecutive series of patients presenting to the Glaucoma Service of a tertiary eye care center that had medically controlled aphakic glaucoma (elevated IOP and typical glaucomatous optic neuropathy, followed by optic nerve head photography or visual field defect in those who were able to take a reliable visual field) following congenital cataract surgery and met the inclusion criteria. The inclusion criteria for group 1 included having a CCT greater than 600 µ and ≤750 µ, being cooperative for tonometry, and lack of nystagmus, corneal edema, corneal scar, or any other corneal pathology such as band shape keratopathy, and not wearing a contact lens.  group 2, normal children group, was selected among normal children coming for a routine eye examination. They had normal ocular exams with a refractive error <±0.5 diopter and no history of eye surgery. 

*Central Corneal Thickness*

All pachymetries were performed on the central cornea with an ultrasound pachymeter (Paxis, Biovision Inc., Clermont-Ferrand, France). Ten measurements were taken in the center of the cornea, and the mean of the readings after omitting the outliers was used as central corneal thickness (CCT). 

*IOP Measurements*

To minimize the potential confounding effects of diurnal variation in IOP, all the study measurements were taken in the same office visit. Measurements were taken in random in order to allow for any variation in IOP caused by applanation. All the patients were examined in a sitting position. The time interval between the tests of each tonometer was about 15 minutes. The GAT and TXL measurements were taken by an experienced glaucoma specialist using a calibrated GAT and TXL, respectively (MRR). Subjects underwent testing with the ORA by a trained nurse. The method of IOP measurement with these tonometers has been described before.^12^^-^^14^ Four to five measurements were taken using the ORA tonometer and the results with the highest waveform score were used for recording CH, CRF, IOPcc, and IOPg values. The average of two IOP measurements by the TXL with inter-measure variability less than 5% was recorded as the TXL values.

*Statistical Analysis*

As the aim of the study was to investigate whether the ORA and TXL measurements matched those of the GAT. Both eyes of the patients were included whenever possible. All the statistical analyses were performed using SPSS for Windows, version 15.0 (SPSS Inc., Chicago, IL). The level of significance was set at P<0.05. 

The current literature suggests that the GAT has superior measurement precision compared with the other available tonometers.^15^ The mean IOP measurements obtained by the ORA and TXL were compared with the measurements obtained by the GAT, using the Student *t *test. All the data are reported as means ± standard deviation. Linear regression analysis was used to evaluate the associations between IOP (as measured by the GAT, TXL, and ORA) and CCT, CH, and CRF. Subsequently, all the independent variables were entered into multiple regression models to assess their relationships with IOP, as measured by the different devices. Bland-Altman plots were constructed to assess the agreement between IOP measurements obtained with the GAT, ORA**, **and TXL; the mean difference and 95% limits of agreement between the devices were calculated. The differences between the measurements for each parameter were plotted against their means.

## Results

**Figure 1:**
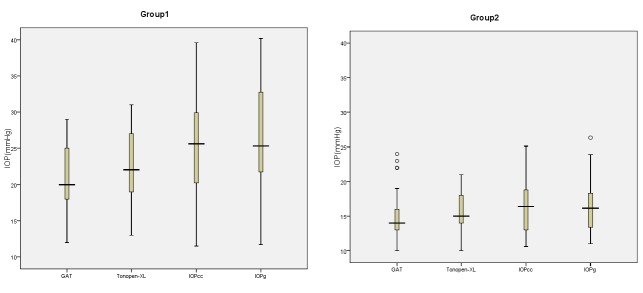
Box-and-Whisker plots, showing mean intraocular pressure (IOP) values obtained by the Goldmann Applanation Tonometer (GAT), Ocular Response Analyzer (ORA), and Tono-Pen XL in groups 1 and 2. IOPcc: Corneal-compensated IOP; IOPg: Goldmann-correlated IOP

This study was conducted on 36 eyes of 23 patients with aphakic glaucoma using 2.02±0.87 anti-glaucoma medications and 40 eyes of 20 age- and sex-matched normal subjects. The demographic data, CCT, CH, and CRF for both groups are shown in table 1. The mean IOP values obtained with each tonometer are illustrated in figure 1 for both groups. The mean±standard deviation of the IOP values obtained by the GAT, TXL, and ORA (IOPcc and IOPg) and the mean difference in IOP measured by the TXL and ORA compared to the GAT in both groups are displayed in table 2. In group 1, the values obtained by the TXL (P=0.004), IOPcc (P=0.005), and IOPg (P<0.0001) were significantly greater than the GAT values. The mean difference for the TXL, IOPcc, and IOPg were 2.1, 6.6, and 7.2 mm Hg, respectively. In other words, the IOP reading by the TXL was closer to the GAT IOP reading in the patients with aphakic glaucoma, and the ORA overestimated IOP compared to the GAT. In group 2, the mean difference of the TXL and IOPcc compared with the GAT was non-significant (0.7 mm Hg, P=0.36 and 1.4 mm Hg, P=0.09, respectively), but the mean difference of IOPg compared with the GAT was statistically greater (1.7 mm Hg, P=0.040). In this group, also the TXL reading was closer to the GAT IOP measurement.

**Table 1:** Demographic data and corneal biomechanical characteristics of groups 1 and 2

	**Group 1**	**Group 2**
Number of eyes	36	40
M/F ratio	14/22	18/22
Age (mean±SD) 95% CI	16.58±5.44 (14.63,18.56)	15.75±5.04 (13.7, 16.80)
Laterality(OD/OS)	21/15	20/20
CCT (μm) (mean±SD) 95% CI	651.1±42 (638.5,671.5)	567.3±32.4 (555.8,577.6)
CH (mean±SD) 95% CI	9.9±3.6 (9.3,11.8)	11±1.8 (10.3,11.6)
CRF (mean±SD) 95% CI	13.2±4.3 (12,15.3)	11.2±2.1 (10.5,11.9)

CCT: Central corneal thickness; CH: Corneal hysteresis; CRF: Corneal resistance factor

**Table 2:** IOP (mm Hg) measured by the Goldmann Applanation Tonometer, Tono-Pen XL, and Ocular Response Analyzer (IOPcc, IOPg) and the mean difference and corresponding P values for IOP measured by different methods compared to the Goldmann Applanation Tonometer in groups 1 and 2

**Tonometer**	**G1**		**G2**	
	**IOP (mm Hg)** **(mean ± SD)**	**Mean difference in IOP compared to GAT and** ** P value**	**IOP (mm Hg)** **(mean±SD)**	** Mean difference in IOP compared to GAT and P value**
GAT 95% CI	20.6±4.6 (18.4, 21.8)	-	14.8±3.7 (13.6,16.1)	-
Tono-Pen XL 95% CI	22.4±4.8 (20.5, 24.3)	2.1 (P=0.004)	15.6±2.5 (14.7,16.4)	0.7 (P=0.36)
ORA IOPcc 95%CI	27.8±12.4 (22, 31.2)	6.6 (P=0.005)	16.1±3.6 (15.1,17.5)	1.4 (P=0.09)
IOPg 95% CI	28.1±10.4 (23.6, 31.2)	7.2 (P<0.001)	16.4±3.8 (15.3,17.9)	1.7 (P=0.04)

G1: Aphakic glaucoma group; G2: Normal children group; GAT: Goldmann Applanation Tonometer; ORA: Ocular Response Analyzer; CI: Confidence interval; IOP: Intraocular pressure; SD: Standard deviation; IOPcc: Corneal-compensated IOP; IOPg: Goldmann-correlated IOP

Table 3 depicts the results of the multiple regression models. One model was developed for each instrument. There was no correlation between CCT and IOP readings with any tonometer. However, in the univariate regression analysis, as is shown in table 4, the IOP measured with all the three tonometers was associated with CCT in group 2 (P<0.05). The only other factor that had a significant association with IOP was CRF in the case of IOPg in both groups (P<0.05). The IOP readings of all the tonometers were associated with CRF and CH in the multiple linear regression analysis. In this model, IOP decreased 1.3 mm Hg/1 mm Hg increase in CH for the GAT, 1.6 mm Hg/1 mm Hg for the TXL, 4.9 mm Hg/1 mm Hg for the IOPcc, and 4 mm Hg/1 mm Hg for the IOPg in group1. These values in group 2 for each mm Hg increase in CH were 1.6, 0.7, 3.5, and 2.8 mm Hg, respectively. In the case of CRF, IOP increased 1.1 mm Hg/1 mm Hg increase in CRF in group 1 for the GAT and 1.2 mm Hg/1 mm Hg in group 2. These figures were 1.4 and 0.7 mm Hg for the TXL, 2.5 and 2.8 mm Hg for IOPcc, and 3 and 3.2 mm Hg for IOPg, respectively. According to these models, all the tonometers seemed to be significantly affected by CH and CRF. The effects of CH and CRF on the measured IOPs were higher in group 2, and the CRF effect was more than that of CH.

**Table 3:** Results of multiple regression analyses for the GAT, Tono-Pen XL, and ORA Tonometers with CCT, CH, and CRF as predictors in groups 1 and 2

**Variables**		**Group**	**Regression model**	**R**	**Adjusted R²**	**Coefficient and P value of CCT**	**Coefficient and P value of CH**	**Coefficient and P value of CRF**
GAT		G1	7.65+0.018CCT-1.383CH+1.125CRF	0.679	0.399	0.175 (0.299)	-1.044 (<0.0001)	1.103 (<0.0001)
		G2	0.629	0.343	0.330 (0.050)	-0.819 (0.004)	0.708 (0.022)	
Tono-Pen XL		G1	23.738-0.006CCT-1.652CH+1.476CRF	0.702	0.438	-0.056 (0.723)	-1.132 (<0.0001)	1.300 (<0.0001)
		G2	0.470	0.152	0.167 (0.369)	-0.592 (0.059)	0.679 (0.051)	
ORA	IOPcc	G1	2.386+0.064CCT-4.918CH+2.506CRF	0.792	0.588	0.216 (0.110)	-1.371 (<0.0001)	0.874 (<0.0001)
		G2	22.547+0.001CCt-3.509CH+2.834CRF	1.000	0.999	0.008 (0.212)	-1.817 (<0.0001)	1.652 (<0.0001)
	IOPg	G1	-5.158+0.050CCT-4.019CH+3.090CRF	0.797	0.598	0.201 (0.131)	-1.331 (<0.0001)	1.280 (<0.0001)
		G2	10.063+0.001CCt-2.822CH+3.296CRF	1.000	0.999	0.008 (0.166)	-1.377 (<0.0001)	1.811 (<0.0001)

G1: Aphakic glaucoma group; G2: Normal children group; GAT: Goldmann Applanation Tonometer; ORA: Ocular Response Analyzer; CCT: Central corneal thickness; CH: Corneal hysteresis; CRF: Corneal resistance factor; IOPcc: Corneal-compensated IOP; IOPg: Goldmann-correlated IOP; SD: Standard deviation

**Table 4:** Comparison of the Goldmann Applanation Tonometer, Tono-Pen XL, and Ocular Response Analyzer IOP values and relations to corneal biomechanical properties as the sole predictor of IOP

		**GAT**	**Tono-Pen XL**	**ORA**	
				**IOPcc**	**IOPg**
Correlation with CCT	G1	0.14 (P=0.41)	0.07 (P=0.69)	0.009 (P=0.96)	0.27 (P=0.11)
	G2	0.44 (P=0.005)	0.32 (P=0.04)	0.38 (P=0.01)	0.52 (P=0.001)
Correlation with CH	G1	-0.04 (P=0.821)	-0.05 (P=0.75)	-0.45 (P=0.008)	-0.10 (P=0.54)
	G2	0.07 (P=0.67)	0.036 (P=0.83)	-0.326 (P=0.04)	0.13 (P=0.41)
Correlation with CRF	G1	0.31 (P=0.08)	0.340 (P=0.05)	0.050 (P=0.77)	0.37 (P=0.02)
	G2	0.27 (P=0.09)	0.24 (P=0.14)	0.16 (P=0.32)	0.59 (P<0.0001)

**Figure 2:**
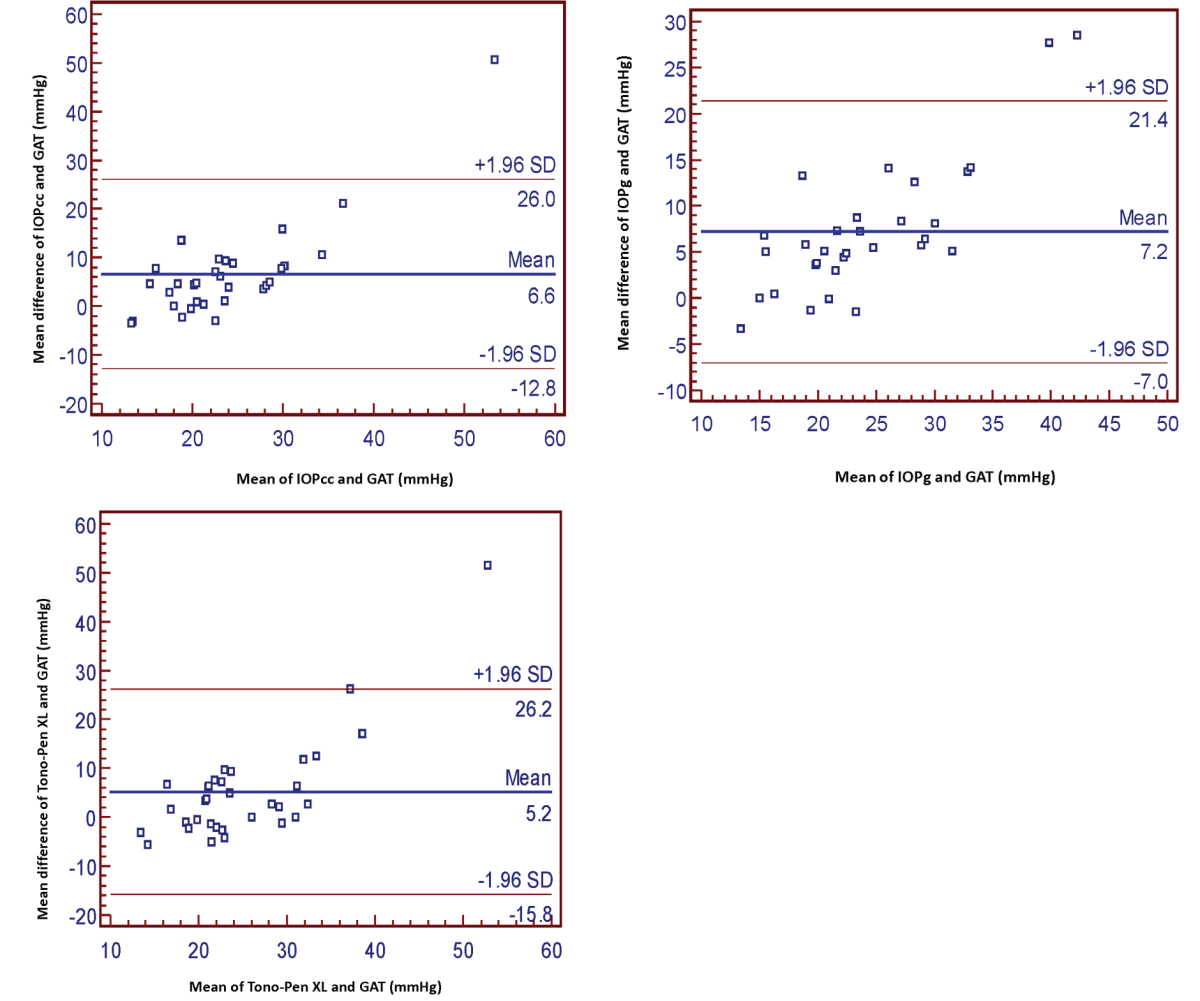
Bland-Altman analysis of intraocular pressure (IOP) measured by the Ocular Response Analyzer (ORA) (IOPcc, IOPg), Tono-Pen XL, and Goldmann Applanation Tonometer (GAT) in group 1. IOPcc: Corneal-compensated IOP; IOPg: Goldmann-correlated IOP; SD: Standard deviation

**Figure 3:**
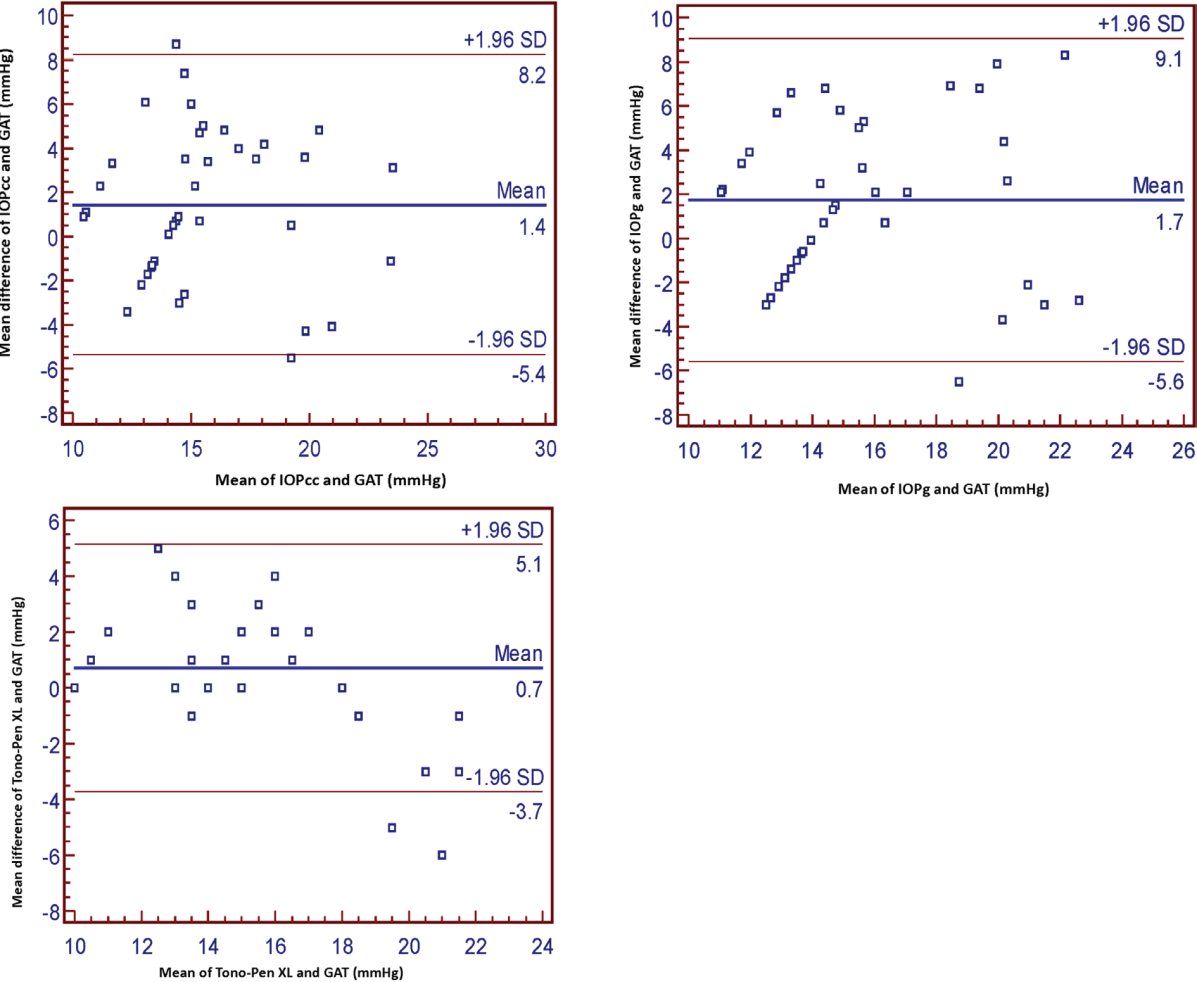
Bland-Altman analysis of intraocular pressure (IOP) measured by the Ocular Response Analyzer (IOPcc, IOPg), Tono-Pen XL, and Goldmann Applanation Tonometer (GAT) in group 2. IOPcc: Corneal-compensated IOP; IOPg: Goldmann-correlated IOP; SD: Standard deviation

G1: Aphakic glaucoma; G2: Control group; GAT: Goldmann Applanation Tonometer; ORA: Ocular Response Analyzer; CCT: Central corneal thickness; CH: Corneal hysteresis; CRF: Corneal resistance factor; IOPcc: Corneal-compensated IOP; IOPg: Goldmann-correlated IOP

figures 2 and 3 display the Bland-Altman plots of the agreement between the TXL, IOPcc, IOPg, and the GAT in groups 1 and 2, respectively. In group 1, the ±1.96 of standard deviations (SD) for all the measurements was greater than that of group 2. In group 1, the ±1.96 SD for IOPcc, IOPg, and the TXL compared to the GAT values was -12.8 to 26.00, -7.00 to 21.4, and -15.8 to 26.2, respectively. These values in group 2 were -5.4 to 8.2, -5.6 to 9.1, and -3.7 to 5.1, respectively. In other words, the values obtained with the ORA and TXL were closer to the GAT values in group 2.

## Discussion

In this study, there was good agreement between the tonometers in group 2, but all the tonometers overestimated IOP in group 1 compared to the GAT. Indeed, in our study, IOPcc and IOPg measurements obtained by the ORA were higher than those obtained by the GAT in both groups. It is a well-known point that noncontact tonometers yield relatively higher measurements compared with the GAT.^16^^,^^17^The difference reached statistically significant levels between the TXL, IOPcc, IOPg, and the GAT in group 1. However, we could not find a similar difference in group 2 except for IOPg. The lack of a good agreement between the tonometers in group 1, contrary to group 2, is clearly evident in the Bland-Altman plots.

Numerous studies have reported that IOPcc is higher than the GAT measurements in both normal and glaucomatous eyes.^13^^,^^18^^,^^19^In the present study, the IOPcc values were 27.8 mm Hg for group 1 and 16.1 mm Hg for group 2, higher than GAT measurements for both groups (20.6 and 14.8 mm Hg, respectively). Although IOPcc was higher than the GAT-IOP in group 2, the difference was not significant statistically. The difference was significantly greater in group 1 compared to group 2 (6.6 vs. 1.4 mm Hg). Hager et al.^20^ showed a mean difference of 1.6 mm Hg comparing IOPcc and GAT in a normal population. Nevertheless, in a group of glaucomatous patients, Martinez-de-la-casa et al.^21^ found a much higher difference between IOPcc and GAT with a mean difference of 8.3±4.0 mm Hg. Because CH did not differ between the groups, CRF may be involved in higher IOPcc readings in the present study.

IOPg values were greater than IOPcc in both groups .The difference between IOPg and IOPcc in group 1 (0.3; 28.1 vs. 27.8 mm Hg) was less than that of group 2 (0.3; 16.4 vs. 16.1 mm Hg). Our results are not in line with the Sullivan-Mee et al.^22^ study, reporting that glaucomatous eyes are characterized by a larger difference between IOPcc and IOPg because IOPcc increases as a result of decreased CH, thus underestimating IOP in glaucomatous eyes. Our contradictory results may be due to higher CRF in group 1. The ultrastructural corneal morphology is the probable cause of greater CRF. CH was lower in group 1 compared to group 2, but not different statistically. It can be concluded that corneal biomechanical properties change in patients with aphakic glaucoma and a thick cornea and that this can be determined by CRF.

Recently, the importance of corneal biomechanical properties, CH, and CRF has been taken into consideration alongside CCT in determining the real IOP. This study found no correlation between the CCT and IOP readings with any tonometer, suggesting the independence of the measured IOP from CCT. This apparently disagrees with most previous studies,^11^^,^^23^^-^^25^ showing a significant dependence of the measured IOP on CCT. However, it is in accordance with the results of the Bayoumi et al.^26^ report. This finding may be related to the fact that the CCT values in the present study were clustered around a specific mean value. In group 1, all the values were more than 600 µ and they did not include thinner corneas. In group 2, the mean CCT was around the mean value for which the GAT was calibrated. In the univariate regression analysis, there was a significant association between CCT and IOP measured with the three tonometers in group 2, which comprised normal subjects with a wider range of CCTs. If the range of CCT was wider in group 1, this result might not have been obtained. In addition, the inclusion of glaucoma patients may confound the association between IOP measurements and CCT because in these participants, IOP may be altered as a result of the disease process. With respect to the results of the multiple regression analysis, CRF was related to the measured IOP; this is consistent with the results of a study by Hager et al.^20^

The present study has some limitations, which must be addressed. There was no independent reference method to assess true IOP to allow us to conclude which method of IOP evaluation was more representative of the true IOP status. To answer this issue, experimental studies involving concomitant manometric and tonometric readings are necessary. Our study also suffers from a limited number of patients. However, this seems to be the first study of its kind, and the rarity of aphakic glaucoma with a thick cornea should be taken into account.

## Conclusion

We believe that, in patients with aphakic glaucoma and a thick cornea, the TXL IOP measurements are closer to the GAT measurements compared to the ORA. Additionally, relying on the result of the ORA, which is proposed to be independent of corneal biomechanical characteristics, may be misleading in this group of patients. Corneal biomechanical properties seem to be changed in this subgroup of patients, which can be determined by CRF. 

The results of our preliminary study need to be supported with larger studies detecting the biomechanical properties of the cornea and agreement between various tonometers in this group of patients. We still are in need of a tonometer to measure IOP independent of the corneal factor, because IOP measurement errors induced by corneal properties can lead to substantial misclassification and possible mismanagement of patients.
